# Lung Cancer Screening for the Poor and Underserved: Should Routine Screening be Performed?

**DOI:** 10.4021/wjon509w

**Published:** 2012-07-05

**Authors:** Vaibhav Verma, Vladimir K. Gotlieb, Joshua Fogel, Alan S. Multz, Geeti Sharma

**Affiliations:** a Nassau University Medical Center affiliated with North Shore/Long Island Jewish Health Care System. Department of Medicine, Division of Hematology and Oncology, 2201 Hempstead Turnpike, East Meadow, NY 11554, USA; b Department of Finance and Business Management, Brooklyn College, Brooklyn, New York, USA; c Indian Army Medical Core, 245 81st Brooklyn, NY, 11209, USA

**Keywords:** Lung cancer, Stage, Screening, Socioeconomic status

## Abstract

**Background:**

Lung cancer is a leading cause of death in United States. A recent study using low dose CT scans for screening long term smokers for lung cancer has, for the first time, demonstrated reduction in mortality, although it is not a standard of care in the community yet.

**Method:**

We analyzed lung cancer data for stages 0 through 4 for 1,412 individuals from, a public hospital, Nassau University Medical Center (NUMC) with patients of lower income, two private hospitals, North Shore University Hospital (NSUH) and Long Island Jewish Hospital (LIJ), with patients of higher income, with average household income per year of 83,795 $, 152,777 $ and 93,234 $ respectively.

**Result:**

Significantly smaller percentages of patients were diagnosed with stages 0 and 1 lung cancer at NUMC (8.55%) versus either NSUH (36.18%, P < 0.001) or LIJ (35.70%, (P < 0.001).

**Conclusion:**

At this point there is evidence that Lung Cancer Screening reduces mortality in long term smokers, but there is debate over, if it should be made into a recommendation. In light of the above study we suggest, that screening for lower socioeconomic class, could be recommended, if not for general population.

## Introduction

Lung cancer is a leading cause of death in the United States, and it accounts for more deaths each year than breast, colon and prostate cancer combined. Screening studies in the 1980’s with chest x rays with or without cytological analysis of sputum did not show any impact on lung cancer specific mortality from screening high risk patients [1, 2]. Although a recent study using low dose CT scans of the chest in patients with higher risk factors for screening demonstrated reduction in mortality [3], this is not the current standard of care in the community. Based on current medical knowledge, only stage I lung cancer has successful cure numbers [4, 5], and a screening method capable of detecting these cases early may have a higher chance, of any method presently available, to be accepted as standard of practice.

We did a study to see if there is a significant difference in the stages of lung cancer at presentation diagnosed in 3 different institutions of the same health system. Geographically located in a 15 mile radius, but represented by different racial, ethnic and socioeconomic groups, these institutions could represent a microcosm of lung cancer. Significant differences would be interesting as it could shed light on current standard practices, those who detect lung cancer earlier or practices that other hospitals are “not doing” and thereby detecting lung cancer later. This could lead to greater mortality and morbidity, because of presentation at later stages of the disease. This analysis could help identify the areas which would benefit from possible screening or other preventive approaches.

## Methods

We obtained retrospective data from 3 hospitals: Nassau University Medical Center-NUMC (years 2000 - 2009), Long Island Jewish Medical Center- LIJ (years 2007 - 2008) and North Shore University Hospital-NSUH (years 2007 - 2008), which are parts of the same health system. We obtained tumor registry summary data for frequency of total lung cancer diagnosed at different stages. The three hospitals serve patients of different economic strata: NUMC patients have an average household income of $ 83,795 per year and average house cost of $ 385,610 and 27% minority population; LIJ patients have an average household income of $ 93,234 per year and average house cost of $ 481,700 and 36% minority population; and NSUH patients have an average household income of $ 152,777 per year, average house cost of $ 999,390 and 20% minority population.

As appropriate, Pearson chi-square analyses were used to compare differences, unless there was a smaller sample size for a cell of less than 5 and where the Fisher’s exact test was used. For the US national data (year 2006), the statistical assumption of independence was slightly relaxed as it is possible that these US data also included the individuals in the NUMC data. Our primary analysis was to compare the percentage for overall stage differences between the hospitals for non-small cell cancer. Stata Version 11 was used for all analyses. All P-values were two sided.

## Results

The eligible sample of those with non-small cell lung cancer included 1,602 individuals. This included 401 individuals from NUMC with 66.83% (n = 268) below age 70 and 33.17% (n = 133) age 70 and above; and 42.4% (n = 170) females and 57.6% (n = 231) males. There were 403 individuals from LIJ with 46.65% (n = 188) below 70 years of age and 53.35% (n = 215) 70 years and above; and 50.62% (n = 204) females, 49.38% and (n = 199) males. There were 798 individuals from NSUH (North Shore University Hospital) with 48.62% (n = 388) below 70 years of age and 51.38% (n = 410) 70 years of age and above and 54.69% (n = 437) females, 45.31% and (n = 361) males. The sample sizes shown in the results section slightly differ for certain analyses due to omission of those with unknown or missing data.

**Figure 1:**
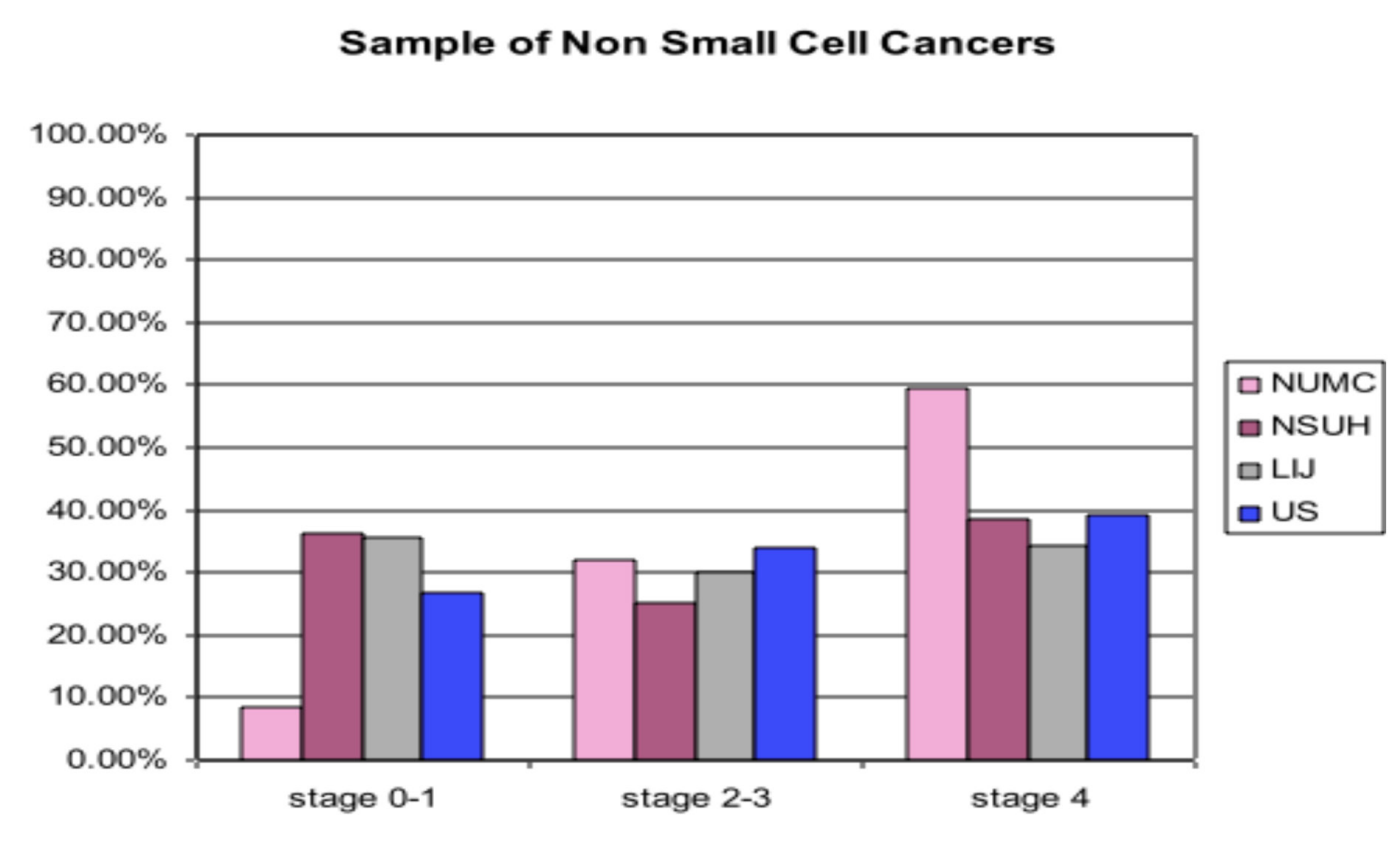
Sample of Non Small Cell Cancers. Vertical axis: percentage of total patients diagnosed in various stages; Horizontal axis: Stage of Lung Cancer; NUMC: Nassau University Medical Center; NSUH: North Shore University Hospital; LIJ: Long Isiand Jewish Hospital; US: United States.

Table 1 and Figure 1 show data for non-small cancers by stage. In the three-level analyses of stage groups of 0-1, 2-3, and 4, NUMC had an overall statistical significance for separate comparisons to NSUH, LIJ, and the US national sample. These analyses showed much lower percentages of stage 0-1 for NUMC as compared to the other sites, mixed results for stage 2-3 with higher percentages for NUMC as compared to NSUH and LIJ but lower than the US national sample and higher percentages for stage 4 as compared to NSUH, LIJ, and the US national sample. The stage 4 percentages for NUMC were at least 20% greater as compared to NSUH, LIJ, and the US national sample (Fig. 1).

**Table 1:** Sample of Non-Small Cell Cancers

Variable	NUMC % (#)	NSUH % (#)	LIJ % (#)	US % (#)	P-value NUMC vs. NSUH	P-value NUMC vs. LIJ	P-value NUMC vs. US
Stage 0-1	8.55% (30)	36.18% (246)	35.70% (136)	26.69% (26,217)	< 0.001	< 0.001	< 0.001
Stage 2-3	31.91% (112)	25.29% (172)	29.92% (114)	34.03% (33,421)			
Stage 4	59.54% (209)	38.53% (262)	34.38% (131)	39.28% (38,579)			
Stage 0-1	8.55% (30)	36.18% (246)	35.70% (136)	26.69% (26,217)	< 0.001	< 0.001	< 0.001
Stage 2-4	91.45% (321)	63.82% (434)	64.30% (245)	73.31% (72,000)			
Stage 0-1	12.55% (30)	48.43% (246)	50.94% (136)	40.46% (26,217)	< 0.001	< 0.001	< 0.001
Stage 4	87.45% (209)	51.57% (262)	49.06% (131)	59.54% (38,579)			

NUMC: Nassau University Medical Center; NSUH: North Shore University Hospital; LIJ: Long Island Jewish; US: United States.

In Table 1, two additional analyses were performed with only two-level analyses of stage groups. One approach grouped stages 2-4 together and the other excluded stages 2-3 from the analyses. In the analyses with stages 2-4 grouped together, NUMC had significantly lower percentages of stage 0-1 as compared to stages 2-4 for separate comparisons to NSUH, LIJ, and the US national sample. This ranged from 18% lower for the US national sample and more than 25% lower than the NSUH and LIJ samples. In the analyses that excluded stages 2-3 from the analyses, NUMC had significantly greater percentages of those with stage 4 as compared to stages 0-1 for separate comparisons to NSUH, LIJ, and the US national sample. This was more than 25% greater than the US national sample and more than 35% greater than the NSUH and LIJ samples.

Table 2 shows data for adenocarcinoma by stage. In the three-level analyses of stage groups of 0-1, 2-3, and 4, NUMC had an overall statistical significance for separate comparisons to NSUH and LIJ. These analyses showed much lower percentages of stage 0-1 for NUMC as compared to NSUH and LIJ, mixed results for stage 2-3 with higher percentages for NUMC as compared to NSUH but lower than LIJ and higher percentages for stage 4 as compared to NSUH and LIJ. The stage 4 percentages for NUMC were at least 30% greater as compared to the NSUH and LIJ samples.

**Table 2:** Sample of Those With Adenocarcinoma

Variable	NUMC % (#)	NSUH % (#)	LIJ % (#)	P-value NUMC vs. NSUH	P-value NUMC vs. LIJ
Stage 0-1	9.90% (10)	45.24% (176)	40.96% (77)	< 0.001	< 0.001
Stage 2-3	24.75% (25)	20.57% (80)	28.19% (53)		
Stage 4	65.35% (66)	34.19% (133)	30.85% (58)		
Stage 0-1	9.90% (10)	45.24% (176)	40.96% (77)	< 0.001	< 0.001
Stage 2-4	90.10% (91)	54.76% (213)	59.04% (111)		
Stage 0-1	13.16% (10)	56.96% (176)	57.04% (77)	< 0.001	< 0.001
Stage 4	86.84% (66)	43.04% (133)	42.96% (58)		

NUMC: Nassau University Medical Center; NSUH: North Shore University Hospital; LIJ: Long Island Jewish; US: United States.

In Table 2, two additional analyses were performed with only two-level analyses of stage groups. One approach grouped stages 2-4 together and the other excluded stages 2-3 from the analyses. In the analyses with stages 2-4 grouped together, NUMC had significantly lower percentages of stage 0-1 as compared to stages 2-4 for separate comparisons to NSUH and LIJ. This was at least 30% lower than the NSUH and LIJ samples. In the analyses that excluded stages 2-3 from the analyses, NUMC had significantly greater percentages of those with stage 4 as compared to stages 0-1 for separate comparisons to NSUH and LIJ. This was more than 40% greater than the NSUH and LIJ samples.

Table 3 shows data for squamous cell carcinoma by stage. In the three-level analyses of stage groups of 0-1, 2-3, and 4, NUMC had an overall statistical significance for separate comparisons to NSUH and LIJ. These analyses showed much lower percentages of stage 0-1 for NUMC as compared to the other sites, higher percentages for stage 2-3 for NUMC as compared to NSUH and LIJ, and higher percentages for stage 4 as compared to NSUH and LIJ. The stage 4 percentages for NUMC were at least 20% greater as compared to NSUH and LIJ.

**Table 3:** Sample of Those With Squamous Cell Carcinoma

Variable	NUMC % (#)	NSUH % (#)	LIJ % (#)	P-value NUMC vs. NSR	P-value NUMC vs. LIJ
Stage 0-1	7.14% (4)	37.88% (50)	39.33% (35)	< 0.001	< 0.001
Stage 2-3	41.07% (23)	36.36% (48)	32.58% (29)		
Stage 4	51.79% (29)	25.76% (34)	28.09% (25)		
Stage 0-1	7.14% (4)	37.88% (50)	39.33% (35)	< 0.001	< 0.001
Stage 2-4	92.86% (52)	62.12% (82)	60.67% (54)		
Stage 0-1	12.12% (4)	59.52% (50)	58.33% (35)	< 0.001	< 0.001
Stage 4	87.88% (29)	40.48% (34)	41.67% (25)		

NUMC: Nassau University Medical Center; NSUH: North Shore University Hospital; LIJ: Long Island Jewish; US: United States. Note: Fisher’s exact test analyses used due to small sample size for NUMC.

In Table 3, two additional analyses were performed with only two-level analyses of stage groups. One approach grouped stages 2-4 together and the other excluded stages 2-3 from the analyses. In the analyses with stages 2-4 grouped together, NUMC had significantly lower percentages of stage 0-1 as compared to stages 2-4 for separate comparisons to NSUH and LIJ. This was at least 30% lower than the NSUH and LIJ samples. In the analyses that excluded stages 2-3 from the analyses, NUMC had significantly greater percentages of those with stage 4 as compared to stages 0-1 for separate comparisons to NSUH and LIJ. This was more than 45% greater than the NSUH and LIJ samples.

## Discussion

We found significant differences for lung cancer where NUMC had greater percentages of stage 4 lung cancer and lower percentages of stages 0-1 lung cancer as compared to the two other local hospitals within the same health care system and the US national data. This was seen in non-small cell cancers as well as adenocarcinoma and squamous cell carcinoma. This is in contrast to an earlier study done in Canada which did not show significant difference in stage at diagnosis in different socioeconomic groups [6]. Apart from this, there are multiple studies assessing impact of socioeconomic status generally on survival of cancer patients [7, 8]

Also, those at NUMC who were diagnosed with lung cancer were relatively younger. This finding was independent of histology. We did not have enough numbers of adenosquamous, large cell carcinoma, carcinoid, and sarcomatoid cancers so we did not compare data for these cancers.

One of the most significant findings of this study was that significantly less people with lung cancer were being diagnosed in stage 1 at NUMC which caters to lower socioeconomic patients and a larger minority population as compared to NSUH and LIJ which caters to predominantly a more affluent section of the population and lesser minority population. This has a very significant bearing on the mortality that lung cancer leads to as for practical purpose stages 0-1 are the only curable stage of lung cancer [5, 6]. This is unfortunate, since despite appropriate diagnostic modalities considerable number of patients become fatal victims of lung cancer because they are financially disadvantaged and therefore may not come in contact with the health system as frequently as they should.

This finding is very relevant in light of the recent study, demonstrating reduction of mortality of lung cancer by low dose CT scan for the first time in 30 years [3]. This has not been made into a formal recommendation yet as debate is going on, considering the costs the health care system would incur to recommend CT scan for screening purpose and possible radiation concerns from CT scans. In view of above findings we want to suggest that screening for lung cancer in people of lower socioeconomic classes or minorities may be useful. This topic should be considered as part of a risk-benefit analysis about screening benefits versus CT dose radiation long term side effects.
